# The effect of manual therapy and stabilizing exercises on forward head and rounded shoulder postures: a six-week intervention with a one-month follow-up study

**DOI:** 10.1186/s12891-019-2438-y

**Published:** 2019-02-18

**Authors:** Kiana Fathollahnejad, Amir Letafatkar, Malihe Hadadnezhad

**Affiliations:** 1 Faculty of Physical Education and Sport sciences, Department of Biomechanics and Sport injuries, Kharazmi University, Tehran, Islamic Republic of Iran; 2 Biomechanics and Corrective Exercise Laboratory, Faculty of Physical Education and Sport sciences, Kharazmi University, Mirdamad Blvd., Hesari St, Tehran, 00982122258084 Iran

**Keywords:** Forward head posture, Function, Manual therapy, Neck pain, Stabilization exercises

## Abstract

**Background:**

The purpose of this study is to evaluate the effect of a six-week combined manual therapy (MT) and stabilizing exercises (SEs), with a one-month follow-up on neck pain and improving function and posture in patients with forward head and rounded shoulder postures (FHRSP).

**Methods:**

Sixty women with neck pain and FHRSP were randomized into three groups: Group 1 performed SE and received MT (*n* = 20), Group 2 performed SE (n = 20) and Group 3 performed home exercises (n = 20) for six weeks. The follow-up time was one month after the post test. The pain, function, and head and shoulder angles were measured before and after the six-week interventions, and during a one-month follow-up.

**Results:**

There were significant within-group improvements in pain, function, and head and shoulder posture in groups 1 and 2. There were significant between-group differences in groups 1 and 2 in head posture, pain, and function favoring group 1 with effect size 0.432(*p* = 0.041), 0.533 (*P* = 0.038), and 0.565(*P* = 0.018) respectively. There were significant between-group differences in both intervention groups versus the control group favoring the intervention groups.

**Conclusion:**

These findings suggest that both interventions were significantly effective in reducing neck pain and improving function and posture in patients. However, the improvement in function and pain were more effective in Group 1 as compared to Group 2, suggesting that MT can be used as a supplementary method to the stabilizing intervention in the treatment of neck pain. More researches are needed to confirm the result of this study.

**Trial registration:**

UMIN000030141 modified on 2018.03.08.

This study is a randomized control trial registered at UMIN-CTR website, the trial was retrospectively registered and the unique trial number is UMIN000030141.

**Electronic supplementary material:**

The online version of this article (10.1186/s12891-019-2438-y) contains supplementary material, which is available to authorized users.

## Background

Forward head posture (FHP) is a kind of poor posture associated with increased kyphosis in the thoracic spine and anterior shoulder position [1]. Poor posture is also associated with altered scapula position in terms of kinematic and muscle activities [2].

In FHP, hyperextension of the upper cervical spine associated with shortening of the upper trapezius, cervical extensor muscles (e.g. Suboccipital, Semispinalis, and splenius), Sternocleidomastoids and the Levator scapulae muscles has been observed [2]. On the other hand, in round shoulder posture (RSP), there is acromion forward displacement in relation to the 7th cervical spinous process and can be measured by the shoulder angle associated with a protracted, anteriorly tilted, internally rotated scapula and the pectoralis minor muscles shortness [3].

Forward head and round-shoulder postures (FHRSP) can result in shoulder pain and dysfunction because of altered scapular kinematics and muscle activity and consequently, placing increased stress on the shoulder [4]. Therefore, FHRSP has to be modified to decrease stress on the shoulder [4].

Changes have been shown in head, shoulder and thoracic posture in people with neck pain compared to asymptomatic ones [5]. Nejati et al. (2014) stated that FHP and thoracic kyphosis are associated with neck pain although they did not find any relationship between RSP and neck pain in Iranian office workers [5]. Similarly, Silva et al. (2008) indicated that those with chronic non-traumatic neck pain have more FHP in standing position than pain-free participants. The difference was considered statistically not clinically meaningful [6].

Despite the fact that neck disorders are common in the population, little evidence supporting effective interventions has been identified [7]. Meanwhile, one of the suggested interventions for improving musculoskeletal disorders is exercise therapy that includes a large variety of methods such as mobilizing, stretching, isometric/static or dynamic strengthening, endurance training, direction-movement control and proprioceptive exercises [7]. Due to the contradictory results from different studies, treatment of neck pain has been considered challenging in clinical healthcare’s [8].

It has been indicated that stabilization exercises (SEs) for the scapula by improving and normalizing muscular activities can improve pain and posture in patients with neck pain and FHP and consequently, the quality of life [9].

On the other hand, manual therapy (MT) is another form of conservative treatment provided by physical therapists, chiropractors, osteopaths and sometimes other healthcare providers. MT can be used as an effective modulation in relieving soft tissue, a range of motion (ROM), and pain, and altering muscle function in musculoskeletal disorders [10].

There is also limited evidence that a multimodal intervention consisting of spinal and neuro-dynamic mobilizations and specific exercises is effective on pain in patients with neck pain [10].

There are studies that have reported considerable effects on improving pain, shoulder and neck ROM, function [11, 12] and posture [9, 13, 14] in patients with FHRSP using exercises, but most of them have no control or comparison groups. To our knowledge, there is no study which compared the effectiveness of a combined MT and SE intervention and stabilizing intervention alone on posture, function, and pain in patients with chronic neck pain. Also, there is no one-month follow-up to support the effect of SE on FHRSP.

Thus, the authors of this study tried to compare the effects of a six-week intervention and one-month follow-up of a combined treatment consisting of SEs and MT, with SE alone, on neck pain, function, and craniocervical and rounded shoulder angles in patients with neck pain and FHRSP.

## Methods

### Participants and procedure

This study is a randomized controlled trial registered at UMIN_RCT website, and the unique trial number is UMIN000030141.

### Ethics and consent statements

The patients were informed about the details of the study and the volunteer subject provided written consent to participate before study enrollment. Informed consent was obtained from all the participants, and procedures were conducted according to the Declaration of Helsinki.

Using a G-power software, with a power of 0.92 and a 3-group design tested at an alpha level of 0.01, 20 subjects per group are required to detect a posture data of 0.8 points (Power and Precision™ 2.0).

This study was conducted for six weeks in the clinic of the university, with a one-month follow-up on married women aged 32–42 years with neck pain and FHRSP. Participants were recruited through physicians, orthopedic surgeons and physical therapists working in private centers.

An experienced physiotherapist assessed the subjects based on clinical history, posture and symptom responses to active movements. For all the assessments, the assessor was blinded to the group allocation. The data analyst was only blinded to the treatment allocation.

Initially, a total of 80 volunteers were evaluated with photogrammetry. The subjects were screened by measuring the cervical angle (CA > 46°) and shoulder angle (SA > 52°) with photogrammetry [15]. Given the inclusion and exclusion criteria, a total of 60 participants were recruited.

The inclusion criteria were non-specific neck pain reproduced by neck movement or provocation tests in the location of the neck [16], neck pain between 3 and 8 cm on a visual analog scale, and at least within the last three months as chronic pain [15, 17–19]. Subjects were excluded when they had a history of cervical spine injury or surgery, neck pain secondary to other conditions (e.g. neoplasm, neurological diseases or vascular diseases), a neurological deficit, infection or inflammatory arthritis in the cervical spine, received physiotherapy within the last 6 months, smoking habits, and poor general health status that would interfere with the exercises [15–18].

The dimension of the sample was calculated to be at least equal to 60 patients (20 per group) based on a 0.95 confidence level, a 0.8 statistical power, and a 0.6 Cohen’s d effect size coefficient.

### Randomization

Randomization was included in 2 sections. Firstly, a list of numbers with each being randomly assigned to a type of treatment (SE or SE plus MT), was provided. Then, the SE or SE plus MT intervention was allocated to each of the participants based on their recruiting order. Secondly, randomization followed a fixed-size design with a concealed allocation ratio of 1:1. Thus, 20 participants were assigned to the SE plus MT intervention, and 20 participants were assigned to the SE program.

Demographic data (age, sex and body mass index (BMI)) and information on smoking habits, physical activities, marital status and referred pain were obtained at baseline. All outcome measures were captured at baseline (time 1), 6 weeks’ post- intervention (time 2), and during one-month follow up (time 3) by an assessor blinded to the group allocation.

In order to reduce bias in data analyses, participants were blinded to which group considered (study flowchart). After randomization, Groups 1 (SE plus MT, *n* = 20) and 2 (SE, n = 20) performed the interventions, 30 min/day, three days/week for six weeks. The subjects in the control group (n = 20) performed only a total of 3 times home exercise program weekly described as a postural correction on daily activities but met more irregularly for lectures and were given information on activities promoting general health. The exercise interventions were performed under the supervision of the physiotherapist and two corrective exercise trainers. The subjects were asked not to receive any extra intervention for neck-shoulder pain. All the subjects could take medications to reduce pain if needed and prescribed by their physicians. However, the participant did not report the use of any pill.

Subjects were also excluded if they missed practice for three consecutive sessions or more. Pain, function and forward head and round-shoulder angles were measured three times in each group at baseline, after a six-week intervention, and during a one-month follow-up after the intervention.

### Instrumentation

#### Pain intensity

To evaluate pain intensity, as one of the primary outcomes, visual analog scale (VAS) was used. This scale is widely used in clinical settings and is known as a valuable tool for assessing pain [18, 19] with ICC = 0.81 [18]. After an explanation, the participants indicated their current pain level by choosing a number from 0 (no pain at all) to 10 (unbearable pain), which were displayed along a horizontal line, 10 cm in length. Each score subjectively reported by a participant was regarded as her level of pain based on a range of scores from 0 to 10 [17].

#### Functional endurance

Progressive Iso-inertial Lifting Evaluation (PILE) has been developed and modified over several years and is known as a standardized protocol and a functional test for measuring muscle endurance with ICC ≥ 0.85 [20, 21]. In addition, the quantification of lifting capacity is an important measure of functional evaluation in this test [21].

Women began with an eight-pound load divided into five-pound iron bar plus container weight. The weight was increased every 20 s at an equal rate to the initial free weight [7, 12, 15, 20]. The subjects performed four lifting movements for 20 s. The PILE protocol (score) included the lifting of weights in a box, in a test for the capability of cervical lifting from waist to shoulder height (30–54 in.). Also, the endpoint was also established when fatigue or aerobic incapability in performing the test were felt [11, 20, 21]. An assessor evaluated the PILE test. To be experienced in the evaluation of the test, the assessor had three days training according to the PILE test protocol.

#### Measurement of forward head and protracted shoulder angles

Posture was assessed using the BioPrint postural analysis system (Biotonix Inc., Montreal, CA). Markers were placed over the right tragus of ear, acromion process and C_7_ spinous process.

Then, the subjects were asked to stand at 40 cm distance in front of a backdrop, bent forward three times, reached overhead three times, and then stood to look straight ahead in their natural resting position. A digital camera (Canon Power shot 95 USA) was placed on a tripod 1 m high and 3.5 m from the wall. Forward head angle (FHA) and forward shoulder angle (FSA) were measured using an image processing software (i.e. Adobe Photoshop) by the respective angles between the centers of the markers. FHA was measured from the vertical anterior to a line connecting the tragus and the C7 marker. FSA was measured from the vertical posterior to a line connecting the C7 marker and the acromial marker. FHA and FSA were measured three times, and the average was used for subsequent photos [15].

Normative value for FSA was an angle greater than 52° and FHP was an angle greater than 46° [1].

### Stabilizing exercises

Neck SEs were performed under the supervision of the physiotherapist and two corrective exercise trainers three times a week for groups 1 and 2. The authors provided cards and written illustrations to inform the subjects how to properly perform the exercises. The neutral posture during daily activities and the exercises was educated using mirrors putting in the side and the front of the subjects. The subjects were asked to have the neutral position on the stable and unstable surfaces during the exercises. The warm up was consisted of 5–6-min walking. Descriptions of the exercises are shown in Tables 1 and 2. Strengthening exercises targeted the periscapular muscles (Y to W, L to W, scapular protraction). Stabilization of the scapula was emphasized during instruction. Strengthening exercises were progressively performed for three sets, with 10 to 15 repetitions. The stretching part of the exercises was done with the purpose of increasing the flexibility of the pectoralis and the cervical neck extensors muscles (pectoralis stretch, chin tuck). Exercises have been approved to be effective on the lengthening of the pectoralis minor, activation of the lower trapezius/middle trapezius, serratus anterior, and improvement of the posture [1].

**Table 1:** Description of strengthening exercises used during 8-week intervention program

Exercise	Description
Y to W	Arms flexed and abducted to 120°, thumbs pointed up, arms 4–5 in. raisied while keeping the retraction of scapula. Then elbows flexed and shoulders moved into a position of extension
L to Y	Arms abducted to 90° and elbows flexed to 90° with retracted scapula and arms externally rotated. Arms raised above the head and fully extended the elbows so that formed the letter Y.
Scapular protraction	Prone hip bridge, shoulder retracted with forearms and toes supporting the body on the floor or table. Then 1–2 cm pushed up while protracting the scapula, and preventing the scapula winging.

**Table 2:** Description of the flexibility exercises used in the 8-week intervention program

Exercise	Description
Pectoralis flexibility	Supine on a foam roller with their spine, contracting transverses abdominous and flattening the lumbar curve against the foam roller. Arms together with shoulders and elbows flexed to 90°, touching forearms and palms. Then shoulders horizontally abducted and scapula retracted, wrists and elbows aligned in the plane of the body. Holded for 5 s and repeated 10 times.
Chin tucks	The chin pushed into the table in an entirely posterior motion. It was not an exercise of tucking the chin to chest through neck flexion

### Manual interventions

Manual therapy interventions were performed by a skilled manual therapist in neck pain based on the study conducted by Gong (2015). Group 1 received manipulation for 10 min, three times a week, for six weeks. The aim of manipulation in the experimental group was to increase flexion, extension, and side bending ROMs by checking the passive motion in cervical facet joints. To assess the passive motions, firstly, the subject was asked to lay supine on the bed while the 7th cervical vertebra was placed on the edge of the bed and others above the 7th vertebra were placed off the bed. Thereafter, a manual therapist held the occipital region and C^6^ spinous process with both hands and checked the mobility of the C5 and C6 joints. This method was used to check the extension ROM restriction of the joint in the cervical spine by holding the C5, C4, and C2 SPs. Then, the therapist applied the manual intervention for extension ROM. To increase flexion ROM, the subject was asked to stay in the same supine position as an extension. The C5–6 joints motion was checked to assess the flexion ROM. Then, the manipulation was performed by checking mobility [22]. To increase the side bending motions of the cervical joints, the same method was used to analyze the ROM restriction and manual application was done for side bending motions [22]. It should be noted that during manual therapy for all extension, flexion and side bending ROMs, movements of the other surrounding joints were prevented (to find full detailed information concerning the applied manual intervention, please check the study of Gong (2015)).

### Statistical analysis

The statistical analyses of the data were performed by a biostatistician using SPSS version 19.0 software (SPSS Inc., Chicago, IL) with the values of *p* < 0.05. Shapiro-Wilk and Levene tests were used to respectively to assess the distribution and homogeneity of variance before performing analysis of covariance (ANOVA). One-way ANOVA was used to identify differences in the VAS, function, and neck and shoulder angles before the interventions among the three groups. Two-way ANOVA was used to evaluate the effects of experimental groups and pre-and post-tests as well as follow-up times on the outcomes. Between-groups effect sizes were calculated and interpreted according to Cohen’s d. Effect sizes were classified as small (d = 0.20), medium (d = 0.50), or large (d = 0.80).

## Results

The results of the Shapiro-Wilk test indicated normal data distribution.

A total of 60 female subjects who had a history of neck pain with forward head and rounded shoulder postures participated in this study (Additional file 1). Table 3 presents the demographic data of all groups.

**Table 3:** Participant characteristics^a^

	Exercise plus manual therapy group	Exercise group	Control group	*P*-value
Age, y	37 ± 3.10	36.4 ± 7.41	36.7 ± 4.38	0.659
Height, cm	165 ± 7.14	170.3 ± 9.09	168.3 ± 9.17	0.431
Mass, kg	63.4 ± 4.32	67 ± 6.13	66.8 ± 5.69	0.385
BMI, kg/m^2^	23.12 ± 1.07	22.32 ± 1.52	22.65 ± 1.44	0.359

^a^Values are mean ± SD. All groups, *n* = 20

There were no differences in the demographic data of all groups. All the subjects completed 6 weeks of exercise intervention with no dropouts.

There were no differences in the characteristics of subjects using VAS, function scores, as well as neck and shoulder angles before the exercise interventions among the three groups (*P* > 0.05) (Table 4).

**Table 4:** Pre-intervention, post-intervention and 1-month follow up values^a^

	Pre-intervention	Post-intervention	1 month follow-up
Exercise plus manual therapy group			
Head posture, degree	47.50 ± 6.00	42.25 ± 4.05	42.58 ± 5.90
Shoulder posture, degree	53.66 ± 1.07	49.95 ± 6.28	49.58 ± 7.08
Function (score)	0.33 ± 0.49	1.50 ± 0.36	1.41 ± 0.41
Pain (0–10)	4.83 ± 0.83	2.16 ± 0.93	1.50 ± 1.08
Exercise group			
Head posture, degree	47.41 ± 1.16	42.75 ± 5.24	42.87 ± 1.15
Shoulder posture, degree	54.00 ± 1.12	49.66 ± 4.72	49.97 ± 5.67
Function (score)	0.25 ± 0.45	1.08 ± 0.19	0.91 ± 0.28
Pain (0–10)	4.91 ± 0.66	3.08 ± 0.79	2.75 ± 0.75
Control group			
Head posture, degree	48.75 ± 0.86	48.16 ± 7.02	47.33 ± 6.88
Shoulder posture, degree	53.25 ± 1.13	52.25 ± 6.28	52.67 ± 6.97
Function (scores)	0.08 ± 0.28	0.16 ± 0.38	0.16 ± 0.39
Pain (0–10)	5.08 ± 0.90	5.11 ± 0.65	5.25 ± 1.21

^a^Values are mean ± SD. All groups, n = 20

### Pain

The ANOVA analysis showed a significant difference in VAS between groups (F = 5.514, *P* = 0.012).

There was within group changes (pretest to posttest) in the VAS score of Groups 1 (2.14 ± 0.1, *P* = 0.008, ES:0.629), and also between Groups 2 (0.70 ± 0.28, *P* = 0.015, ES: 611), but no difference in the control group (*P* = 0.18) (Tables 4 and 5).

**Table 5:** Change scores (post-intervention-pre-intervention; 1 month follow up-post-intervention) *

	Differences from Post-intervention to pre-intervention		
	Exercise plus manipulation group	Exercise group	Control group
Head posture, degree	−5.31 ± 0.41¤	−5.21 ± 0.18¤	−1.08 ± 0.75
Shoulder posture, degree	−5.33 ± 1.08¤	−5.48 ± 1.33¤	−1.25 ± 0.43
Function (scores)	1.25 ± 0.21*¤	0.95 ± 0.32¤	0.07 ± 0.12
Pain (0–10)	−2.55 ± 0.41*¤	−1.89 ± 0.13¤	0.41 ± 0.21
Differences from 1 month follow up to post-intervention			
Head posture, degree	0.65 ± 0.22	0.54 ± 0.31	−1.23 ± 1.02
Shoulder posture, degree	−1.16 ± 0.42	0.67 ± 0.11	0.48 ± 0.32
Function (scores)	−0.32 ± 0.13	− 0.47 ± 0.18	0.18 ± 0.14
Pain (0–10)	−1.34 ± 0.22¥	−0.75 ± 0.21	0.25 ± 0.22

A positive change score indicates an increase in values

A negative change score indicates a decrease in values

¤Statistically significant difference from the control group (*P* < .05)

¥Statistically significant difference from 1-month follow-up to post-intervention (*P* < 0.05)

*Statistically significant difference from exercise group (*P* < 0.05)

*Values are mean ± SD

There was a significant difference in VAS in the pre- and post-tests in Groups 1 and 2 (*p* = 0.038).

There was a significant difference in VAS from a 1-month follow-up to post-intervention (decrease, − 1.34 ± 0.22 score change) only in the exercise plus MT group (*P* = 0.016) (Table 5).

### Function

The ANOVA analysis showed a significant difference in function between the groups (F = 5.213, *P* = 0.009).

There was within group changes (pretest to posttest) in the head posture of Group 1 (pretest: 0.33 ± 0.49, posttest: 1.50 ± 0.36, and differences: 1.25 ± 0.21, *p* = 0.002, ES:0.638) and Group 2 (pretest: 0.25 ± 0.45, posttest: 1.08 ± 0.19, and differences: 0.95 ± 0.32, *P* = 0.005, ES:0.608). There was no significant within group changes in Group 3 (pretest: 0.08 ± 0.28, posttest: 0.16 ± 0.38, and difference: 0.07 ± 0.12, p: 0.23) (Tables 4 and 5).

There was a significant between group post-test difference in function in Groups 1 and 2 (*p* = 0.018).

There was no significant difference in function from a 1-month follow-up to post-intervention in all the groups (*P* = 0.128) (Table 5).

### Head posture

The ANOVA analysis showed that there was significant difference in head posture between the groups (F = 4.312, *P* = 0.016).

There was within group changes (pretest to posttest) in the head posture of Groups 1 (pretest: 47.50 ± 6.00, posttest: 42.25 ± 4.05, and differences: − 5.31 ± 0.41, *p* = 0.001, ES: 0.721) and Groups 2 (pretest: 47.41 ± 1.16, posttest: 42.75 ± 5.24, and differences: − 5.21 ± 0.18, *P* = 0.003, P:0.714). There was no significant within group changes in Groups 3 (pretest: 48.75 ± 0.86, posttest: 48.16 ± 7.02, and differences: 1.08 ± 0.75, *P* = 0.62) (Tables 4 and 5).

There was a significant difference in head posture degree between pre- and post-tests in Groups 1 and 2 (*p* = 0.041).

There was no significant difference in head posture from a one-month follow-up to post-intervention in all the groups (*P* = 0.148) (Table 5).

### Shoulder posture

The ANOVA analysis showed that there were significant differences in shoulder posture between the groups (F = 4.318, *P* = 0.018).

There was within group changes (pretest to posttest) in the head posture of Groups 1 (pretest: 53.66 ± 1.07, posttest: 49.95 ± 6.28, and differences: − 5.33 ± 1.08, *P* = 0.006, ES: 0.597) and Groups 2 (pretest: 54.00 ± 1.12, posttest: 49.66 ± 4.72, and differences: − 5.48 ± 1.33, *P* = 0.004, ES:0.619). There was no significant within group changes in Groups 3 (pretest: 53.25 ± 1.13, posttest: 52.25 ± 6.28, and differences: − 1.25 ± 0.43, *P* = 0.19) (Tables 4 and 5).

There were no differences among the shoulder posture degree of Groups 1 and 2 (*P* = 0.54).

There was no significant difference in shoulder posture from a one-month follow-up to post-intervention in all the groups (*P* = 0.213) (Table 5).

## Discussion

This study aimed to investigate if SEs plus MT is more effective than SEs alone in the management of neck pain.

The results showed that pain and function significantly improved in the treatment groups after the six-week exercises; this improvement was also maintained after one-month follow-up. Moreover, when compared with Group 2, function and pain improvement in Group 1 were more effective.

Altered scapula-humeral rhythm and decreased upward rotation of the scapula have been seen in individuals with FHRSP, fatigue, and disability in shoulder muscle, impingement or instability of the glenohumeral joint [23].

The trapezius and serratus anterior muscles as the upward rotators of the scapula are essential for normal shoulder function [24]. In this condition, the middle part of the trapezius muscle helps just to control the amount of abduction of the scapula during upward rotation and does not play any role in the upward rotation of the scapula [25].

In this study, L to Y exercise was selected because it can externally rotate the shoulder to end range at 90° of abduction and cause maximum scapular depression, leading to increased activity in the lower trapezius muscle [1, 11]. But there is low evidence of high activation of the upper trapezius muscle during L to Y exercise without resistance to the head and neck [22].

Gong (2015) demonstrated high levels of EMG activity in the serratus anterior muscle during EMG studies at 90° flexion with scapular protraction (Y to W). The ‘L to W″ and “L to Y” exercises, with the shoulder horizontally abducted with external rotation and with the arm raised overhead in line with the lower trapezius muscle fibers, with the subject in the prone position, generated the highest level of mean EMG activity in the middle part of the trapezius muscle [22]. Therefore, by performing the two exercises in this study, it can be speculated that improvement in pain and function may be caused by a change in motor recruitment, or strengthening of the trapezius and serratus anterior muscles.

Studies supported the present results considering pain reduction and function improvement through MT [10, 26, 27]. Hakkinen et al. (2007,) which showed that both MT and stretching were effective for short-term treatments to reduce both spontaneous and strain-evoked pain in patients with chronic neck pain [26]. Howe et al. (1983) considered medication and manipulation intervention for their subjects separated into two groups. The group that received manipulation had an immediate improvement in ROM and relief from pain after treatment [28].

Therefore, in the current study, it can be inferred that the statistical advantages of Group 1 as compared to Group 2 in function improvement and pain reduction over the six-week intervention and the one-month follow-up, can be due to improved tissue extensibility and ROM; relaxation; altered muscle function, and reduction of soft tissue swelling and inflammation using MT [10, 26, 27]. Also, it is possible that the decrease in pain reduced inhibition of the motor system and in part improved function [28].

The results of this study indicated that cervical and shoulder angles significantly decreased, suggesting that both interventions showed statistically significant effects on cervical and shoulder angles. Interestingly, the differences were maintained after a one-month follow-up. The control group did not demonstrate such improvement of posture. However, there were no differences between experimental groups in improvement of posture following the interventions. Exercise interventions aimed at strengthening weak muscles and stretching tight ones. It is thought to improve FHP and RSP [1]. Im et al. (2016) reported that stabilization training can improve the control of the serratus anterior and upper trapezius muscles, and bring the scapular and thoracoscapular closer to normal positions from FHP [9]. Lynch et al. (2010) reported that the exercise intervention (stretching of the anterior shoulder muscles and strengthening of the posterior shoulder muscles) considerably improved FHRSP in elite swimmers [1]. Also, Ruivoet al. (2016) successfully improved FHP and protracted shoulder posture following a 32-week resistance and stretching training. The authors suggested that the training used in this study seems to strengthen the scapular stabilizers and stretch the pectoralis minor, resulting in decreased cervical and shoulder angles to improve RSP [14].

The results indicated that a combined treatment consisting of MT and SE is more effective than SE alone in the management of chronic neck pain: it reduces chronic pain and improves posture, and upper limb function.

### Limitations

Due to the small sample size, the data analysis of this study can not be considered powerful enough to determine the real differences between groups. Randomized controlled clinical trials with large sample size are needed to confirm the efficacy of the combination of SEs and MT in patients with FHP and RSP.

## Conclusion

In this study, it was found that a combined treatment consisting of MT and SEs, performed three times a week over a 6-week period by women aged 32–42 years with neck pain, and FHRSP, resulted in pain reduction, and posture and function improvements, with a reduction in cervical and shoulder angles. The findings showed that improvement in function was statistically more effective in Group 1 than Group 2. The results of the study also demonstrated that from posttest to one-month follow-up period outcomes maintained specially in Group 1. However, further studies on large populations are required to establish the ultimate effectiveness of SEs plus MT.

Since disability is usually accompanied by a substantial effect on daily life and results in an extensive use of healthcare resources [27], it is suggested to investigate the effect of MT plus SEs on disability of patients with chronic neck pain. Also, to better understand how upper limb function is modified after the MT plus stabilizing exercises, further studies are needed to provide sufficient or reliable information on the recruitment of all the muscles involved in functional movement using electromyography.

## Additional file

Additional file 1: Study flowchart. Flow Diagram. Participants and study design in allocation, enrollment, pre-test, post-test, and follow-up provided in consort flowchart. (DOCX 44 kb)

## Abbreviations

BMI: Body mass index
FHA: Forward head angle
FHP: Forward head posture
FHRSP: Forward head and rounded shoulder postures
FSA: Forward shoulder angle
MT: Manual therapy
PILE: Progressive Iso-inertial Lifting Evaluation
ROM: Range of motion
RSP: Round shoulder posture
SEs: Stabilizing exercises
