# Editorial expression of concern: The effect of manual therapy and stabilizing exercises on forward head and rounded shoulder postures: a six-week intervention with a one-month follow-up study

**DOI:** 10.1186/s12891-025-09260-0

**Published:** 2025-10-29

**Authors:** Kiana Fathollahnejad, Amir Letafatkar, Malihe Hadadnezhad

**Affiliations:** 1 Faculty of Physical Education and Sport sciences, Department of Biomechanics and Sport injuries, Kharazmi University, Tehran, Islamic Republic of Iran; 2 Biomechanics and Corrective Exercise Laboratory, Faculty of Physical Education and Sport sciences, Kharazmi University, Mirdamad Blvd., Hesari St, Tehran, 00982122258084 Iran

**Correction: BMC Musculoskelet Disord 20, 86 (2019)**

**https://doi.org/10.1186/s12891-019-2438-y; Published: 18 February 2019**

The Editor is issuing an editorial expression of concern to alert readers that concerns have been raised regarding the ethical approval for this research [1]. The authors have provided conflicting documentation regarding which Institutional Review Board approved their work, the approval numbers and the dates at which these approvals were received.

Amir Letafatkar does not agree to this editorial expression of concern. Kiana Fathollahnejad and Malihe Hadadnezhad have not responded to any correspondence from the editor about this editorial expression of concern.
