# Supplementary material for: The effect of manual therapy and stabilizing exercises on forward head and rounded shoulder postures: a six-week intervention with a one-month follow-up study
Source: BMC Musculoskelet Disord. 2019 Feb 18;20:86. doi: 10.1186/s12891-019-2438-y (PMC6379958; doi:10.1186/s12891-019-2438-y)
Supplement: Supplementary file 1 — Study flowchart. Flow Diagram. Participants and study design in allocation, enrollment, pre-test, post-test, and follow-up provided in consort flowchart. (DOCX 44 kb) [file 12891_2019_2438_MOESM1_ESM.docx]

**CONSORT Flow Diagram**

Excluded (n= 20)

•  Not meeting inclusion criteria (n= 20)

• Declined to participate (n= 0)

•  Other reasons (n= 0)

Lost to follow-up (give reasons) (n= 0) Discontinued intervention (give reasons) (n= 0)

Lost to follow-up (give reasons) (n= 0) Discontinued intervention (give reasons) (n= 0)

Lost to follow-up (give reasons) (n= 0) Discontinued intervention (give reasons) (n= 0)

Allocated to stabilizing exercises plus manual therapy (n= 20)

• Received allocated intervention (n= 20)

Allocated to control group (n= 20)

• Received allocated intervention (n= 0)

Allocated to stabilizing exercises (n= 20)

• Received allocated intervention (n= 20)

Randomized (n= 60)

Analysed (n= 20)
• Excluded from analysis (give reasons) (n= 0)

Analysed (n= 20)
• Excluded from analysis (give reasons) (n= 0)

Analysed (n= 20)
• Excluded from analysis (give reasons) (n= 0)

Assessed for eligibility (n= 80)

## Enrollment

## Allocation

## Post test

## Follow- up
